# Proto-Tethyan tectonics in East China: a revisit

**DOI:** 10.1093/nsr/nwaf153

**Published:** 2025-04-21

**Authors:** Qing-Ren Meng, Guo-Li Wu, Bo Wan, Ri-Xiang Zhu, Liang Duan, Bin Wang, Jian-Min Hu

**Affiliations:** State Key Laboratory of Lithospheric and Environmental Coevolution, Institute of Geology and Geophysics, Chinese Academy of Sciences, Beijing 100029, China; College of Earth and Planetary Sciences, University of Chinese Academy of Sciences, Beijing 100049, China; Key Laboratory of Paleomagnetism and Tectonic Reconstruction, Ministry of Narural Resources, Institute of Geomechanics, Chinese Academy of Geological Sciences, Beijing 100081, China; State Key Laboratory of Lithospheric and Environmental Coevolution, Institute of Geology and Geophysics, Chinese Academy of Sciences, Beijing 100029, China; College of Earth and Planetary Sciences, University of Chinese Academy of Sciences, Beijing 100049, China; State Key Laboratory of Lithospheric and Environmental Coevolution, Institute of Geology and Geophysics, Chinese Academy of Sciences, Beijing 100029, China; College of Earth and Planetary Sciences, University of Chinese Academy of Sciences, Beijing 100049, China; State Key Laboratory of Continental Dynamics and Department of Geology, Northwest University, Xi'an 710069, China; State Key Laboratory of Continental Dynamics and Department of Geology, Northwest University, Xi'an 710069, China; Key Laboratory of Paleomagnetism and Tectonic Reconstruction, Ministry of Narural Resources, Institute of Geomechanics, Chinese Academy of Geological Sciences, Beijing 100081, China

**Keywords:** Proto-Tethys, North Qinling, West Cathaysia, Wuyi–Yunkai orogen, Fuxi–Nüwa orogen

## Abstract

Two major Early Paleozoic orogens exist in East China, the North Qinling–North Tongbai and Wuyi–Yunkai orogens, which used to be regarded as two independent systems controlled by distinct plate tectonic processes. The North Qinling–North Tongbai orogen consists predominantly of subduction- and collision-related rock assemblages, whereas the Wuyi–Yunkai orogen is made up chiefly of collision-related foreland fold-thrust systems. It is demonstrated that the two seemingly unrelated orogens are actually two components of an immense single orogen that is here named the Fuxi–Nüwa orogen. We stitch together a complete picture of the Fuxi–Nüwa orogen based on a holistic treatment of Early Paleozoic tectonics in East China. It is surmised that the Fuxi–Nüwa orogen was created by initial continental promontory–intraoceanic arc point collision and final complete collision of the North China block with West Cathaysia. The orogen was then torn apart in the Carboniferous, with the Wuyi–Yunkai orogen gradually moving away along a crustal-scale sinistral shear zone. Our proposed tectonic panorama offers satisfactory explanations for many perplexing questions that have plagued geologists for decades, such as the tectonic driver for the Kwangsian orogeny in West Cathaysia, mechanisms for ultra-high-pressure metamorphism and exhumation of the eclogized continental rocks in the North Qinling, simultaneousness of Silurian high-flux magmatism in the North Qinling–North Tongbai and Wuyi–Yunkai orogens, broad absence of Silurian–Early Devonian strata, and causes for Mid–Late Devonian marine inundation over West Cathaysia. Discovery and restoration of the Fuxi–Nüwa orogen will reshape our understanding of tectonic evolution of the Proto-Tethyan regimes, and necessitate reassessment of potentials of oil-gas resources in East China.

## INTRODUCTION

**Figure 1.**
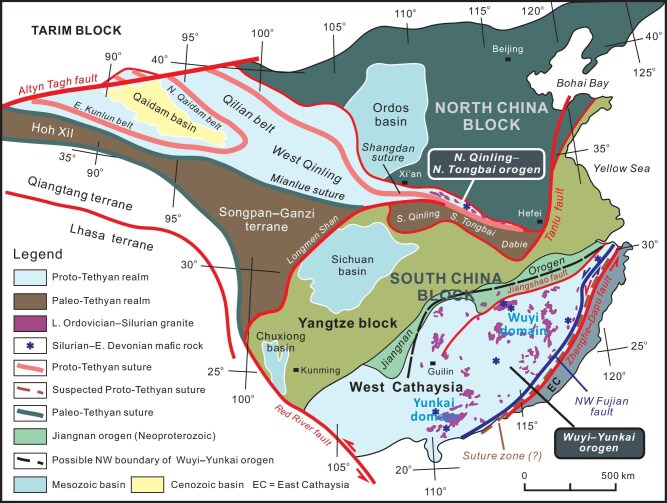
Tectonic map showing main tectonic elements and traces of the Proto-Tethyan sutures in China. The North Qinling–North Tongbai orogen and Wuyi–Yunkai orogen are located at the southern edge of the North China block and the southeastern portion of the South China block, respectively, and usually taken as two independent orogens. Note the occurrences of Late Ordovician–Silurian granite and Late Silurian–Early Devonian mafic rocks in the North Qinling–North Tongbai and Wuyi-Yunkai orogens. The Zhenghe–Dapu or Northwest Fujian fault separates West from East Cathaysia.

**Figure 2.**
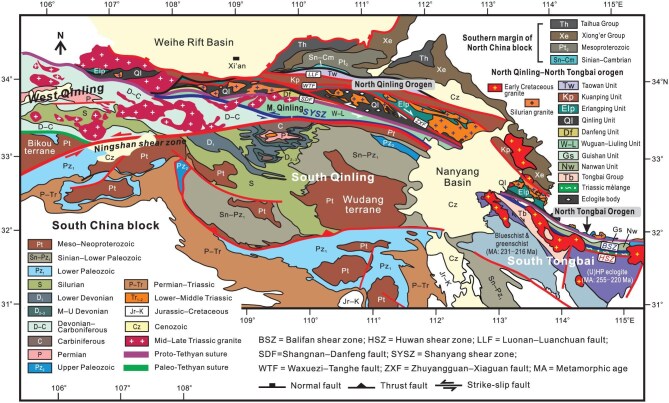
Geologic map of the Qinling–Tongbai orogen, showing distribution of main tectonic elements and main fault zones. Note that all the tectono-petrologic units in the North Qinling and North Tongbai orogens are in fault contact and most of the faults possess pronounced strike-slip components. The Shangdan fault separates from the Middle Qinling from the North Qinling, and the Shanyang–Balifan shear zone serves as the boundary between the Middle Qinling and the South Qinling/South Tongbai domain. Also noticeable is the distribution of Early Paleozoic eclogized bodies that exclusively occur in the Qinling Unit. Silurian granites are merely present in the North Qinling and North Tongbai orogens and absent in the region south of the Shanyang shear zone.

The continental region of East China formed as a consequence of two-stage amalgamation of the North and South China blocks in the Early Paleozoic and Triassic, respectively, and two sutures, the Shangdan and Mianlüe sutures, record the closure of the Proto- and Paleo-Tethyan oceans [1]. The Proto-Tethyan Shangdan suture in the North Qinling extends to the west and is linked with suture in the Qilian belt (Fig. 1). Diverse models have been put forward to explain the tectonic evolution of the Qinling–Tongbai orogenic belt [1–8], reflecting divergent opinions on development of the orogen. The North Qinling–North Tongbai orogen (NQNTO) boasts a variety of geologic records of Early Paleozoic tectonics, which make it an exceptional region to address several outstanding questions related to the tectonic history of the Proto-Tethys in East China. Multiphase deformations occurred in the North Qinling during Paleozoic and Mesozoic times [9–11]. Strike-slip faulting of various scales and ages profoundly affected the NQNTO, resulting in significant distortion and fragmentation of its Early Paleozoic tectonic framework [12,13]. The present configuration of the whole Qinling–Tongbai–Dabie orogen thus informs us of a blurred vision of its original framework (Fig. 2). It remains a great challenge to restore Early Paleozoic history of the NQNTO, such as subduction–collision processes and the resulting architecture [7]. Existing tectonic models assume that the Early Paleozoic NQNTO resulted from the collision of the South Qinling with the North China block (NCB) along the Shangdan suture [1,3]. Ophiolitic complexes and associated rocks represent remnants of the Proto-Tethyan oceanic crust, volcanic arc, and backarc basin in an active continental margin [14]. Previous studies have undoubtedly enriched our understanding of the Qinling orogen in many respects and led to accumulation of a great mass of data. However, quite a few questions remain unanswered about the Proto-Tethyan tectonics of the NQNTO, including subduction polarity, timing of collision, role of late strike-slip faulting, and tectonic framework.

The South China block (SCB) comprises the Yangtze and Cathaysia blocks (Fig. 1), and its tectonic evolution has been extensively investigated and well documented [15–18]. The two tectonic elements were amalgamated ∼815 Ma during the assembly of the Rodinia supercontinent along the Jiangnan orogenic belt [17]. Diverse models have been proposed to illustrate the Neoproterozoic tectonics of the SCB [19], arguing that the Jiangnan orogen experienced complicated volution involving development of intraoceanic arcs and backarc basins as a result of either unidirectional or bidirectional subduction of the South China oceanic plate [18,20]. The Jiangshan–Shaoxing fault zone roughly follows the suture between the Yangtze and Cathaysia blocks on account of the presence of ophiolitic assemblages [21]. The Cathaysia block is dividable into the western and eastern parts that are separated by the Zhenghe–Dapu fault [22]. West Cathaysia experienced Early Paleozoic crustal shortening, metamorphism and magmatism, and was further affected by Early Triassic (Indosinian) and Late Mesozoic (Yanshanian) deformations and magmatism [23]. In contrast, East Cathaysia is predominated by late Mesozoic igneous rocks, and contains sparse Paleozoic geologic records. Implicitly, West and East Cathaysia had not been juxtaposed until the Mesozoic. Li *et al.* [24] renamed West Cathaysia as the Wuyi–Yunkai orogen (WYO), which has now gained wide acceptance. The WYO covers both West Cathaysia and the Jiangnan orogenic belt, and possibly extends northeastward to the Korean Peninsula and SW Japan and southwestward to Indochina [25]. In addition, the WYO is taken as either an intraplate [24,26] or a collisional orogen [27,28]. An angular unconformity separates lower from upper Paleozoic strata in the WYO, and the crustal shortening event responsible for the stratigraphic discordance was termed as the Kwangsian Movement [29].

The NQNTO and WYO have long been taken as two independent Early Paleozoic orogens. Our recent investigations, however, reveal that the two orogens are in reality two components of a single immense orogen, which is named here the Fuxi–Nüwa orogen. This study is poised to rebuild the tectonic history of the Fuxi–Nüwa orogen by piecing together the dismembered parts that had been displaced by large-magnitude sinistral strike-slip faults since the late Paleozoic.

## THE NORTH QINLING–NORTH TONGBAI OROGEN

### The North Qinling

The North Qinling consists of five units, which are, from north to south, the Taowan, Kuanping, Erlangping, Qinling, and Danfeng units, respectively, and all the units are in fault contact (Fig. 2). The Taowan Unit is Ordovician in age and separated by two angular unconformities from the Cambrian and Permian [30]. It consists mainly of phyllite and carbonaceous slate, and its complex deformation is thought to result from both sinistral transpression of the Luoluan fault on the south [31] and Mesozoic south-directed thrusting and folding [32].

The Kuanping Unit is composed of plagioclase amphibolite, greenschist, paraschist, gneisses and marble and divided into three parts separated by faults or angular unconformities. The Lower Kuanping is dominated by meta-mafic rocks with N-MORB, E-MORB or OIB affinities, yielding zircon U-Pb ages of 1450–950 Ma [33,34]. Meta-mafic rocks are considered as fragments of oceanic crust, representing late Neoproterozoic ophiolite in the aftermath of closure of the Kuanping Ocean [33,34]. The Middle Kuanping (the Sichakou Formation) comprises primarily paraschist and quartzite, and detrital zircons give youngest ages from 578 to 500 Ma [35]. The Upper Kuanping (the Xiewan Formation) consists predominately of marble and some paraschist, detrital zircons of which yield ages as young as 418–380 Ma [35]. Given it underwent metamorphism from 370 to 320 Ma [35], the Upper Kuanping Unit should be of Devonian age.

The Erlangping Unit is composed of basic to intermediate metavolcanic, metasedimentary and intrusive rocks, including gabbros, basalts, pyroclastics, basic–intermediate intrusives, and fine-grained siliciclastics. The closely associated volcanic and sedimentary rocks, together with their geochemical similarities, are interpreted as products of intra-oceanic arcs [4,36,37] or backarc basins [1,3]. The Erlangping Unit was intruded by 488–470 Ma granitoids, indicating that it should be older than ∼490 Ma [4]. It is worth noting that the Erlangping subduction-related magmatism and sedimentation might have persisted in the Late Ordovician in adjacent regions such as the northern West Qinling [38] and the North Tongbai orogen [39]. Mafic and granitic magmatism occurred during the Late Ordovician and Silurian, with resulting rocks intruding the Erlangping, Qinling and Danfeng units [40,41].

The Qinling Unit manifests itself as a narrow wedge-shaped tectonic sliver, pinching out to the east in the North Tongbai (Fig. 2). Protolith ages of the metamorphic assemblage range from 900 to 800 Ma [4]. Three phases of metamorphism are identified, progression at 500–490 Ma and retrogression at 480–460 Ma and 440–420 Ma, respectively [42–44]. The Qinling Unit is bounded on the north by the Zhuxia fault and on the south by the Shangdan fault, and the two faults are in practice right-slip and left-slip shear zones, respectively (Fig. 2). Pervasive ductile deformation characterizes the Qinling Unit, with well-developed SE-plunging low-angle stretching lineation. Ductile deformation is attributed to the westward lateral extrusion of the Qinling Unit during the period from ∼427 to 400 Ma [45,46]. Xu *et al.* [47], however, argued that the extensive ductile deformation was related to subduction-related thermal doming and supracrustal extension of the Qinling arc terrane in the Silurian. Detailed geological mapping and structural analysis suggest that the whole Qinling Unit must have undergone orogen-parallel extension in the middle Paleozoic, and the resulting low-angle detachment surface was then folded in the late stage [48]. Plausibility of this interpretation is supported by field observations that reveal many dextral and sinistral shear zones with low-angle stretching lineations in the Qinling Unit, which in reality represent the limbs of tight folds of the early extensional detachment faults.

The Danfeng Unit consists of amphibolites, serpentinites, and schists, and is commonly interpreted as a subduction-related ophiolitic complex, called the Danfeng or Shangdan mélange [3,49]. However, the Danfeng mélange has seldom been structurally studied, compared with the typical mélanges documented in the Qilian Shan and other orogens [50,51]. Its protoliths include pillow basalts, gabbro, peridotite, pyroclastics, and greywacke, and they all experienced greenschist- and amphibolite facies metamorphism. The timespan of the Danfeng Unit is from the Cambrian to Ordovician based on both radiolarian ages [52] and U-Pb zircon dating [3]. The popular views hold that the Danfeng Unit resulted from the Proto-Tethyan plate subduction beneath the Qinling Unit [3,10], whereas other studies interpret the Danfeng Unit as the products of backarc basins [53]. Late Silurian–Early Devonian mafic rocks intrude the Danfeng Unit, such as the Lajimiao and Sifangtai mafic complexes [54].

### Middle Qinling

The Middle Qinling comprises the Wuguan and Liuling units. The Wuguan Unit is a narrow zone sandwiched between the Danfeng and Liuling units (Fig. 2). It consists of metasedimentary rocks that all experienced amphibolite facies metamorphism. Field investigations allow us to redefine the Wuguan Unit as a ductile shear zone resulting from sinistral transpression. Detrital zircons show a major peak at 462 and subordinate peaks at 828 and 446 Ma [55]. Also noticeable is the age spectrum of detrital zircons from the Wuguan shear zone, which coincides with that of the Liuling Unit and exhibits a major peak at ∼460 Ma and a subordinate peak from 786 to 927 Ma [56]. The youngest ages of detrital zircons are ∼358 Ma [57], suggesting that this unit is as young as the Early Carboniferous. Amphibolites in the Wuguan sinistral shear zone yields metamorphic zircon ages of 321–318 Ma and hornblende gives ^40^Ar/^39^Ar ages of 306–299 Ma [55,58]. Implicitly, sinistral ductile shearing must have initiated since the Late Carboniferous.

The Liuling Unit is dominated by clastic rocks, and assigned to be Middle–Late Devonian in age [59]. It is bounded on both the north and south by ductile shear zones, respectively. The contact between the Liuling Unit and underlying rocks is either an angular unconformity or a fault [59]. Detrital zircons of sandstones yield three age clusters at 500–400 Ma, 850–700 Ma, and 1000–900 Ma [56]. The first group of detrital zircon ages can be further divided into three age peaks at ∼497 Ma, ∼451 Ma, and ∼420 Ma, which are in accord with three distinct phases of magmatism in the North Qinling orogen [41]. Of particular importance is the presence of detrital metamorphic zircons from eclogites [56]. Zircon grains aged 1000–900 Ma undoubtedly came from the Qinling Unit that possesses early Neoproterozoic granitic intrusives with an age range from 918 to 914 Ma [56]. It is worth noting that Devonian strata south of the Shanyang fault or in the South Qinling exhibit sedimentary facies sequence that is completely different from that of the Liuling Unit [60].

### The North Tongbai

The Tongbai orogen is separated from the North Qinling orogen by the Nanyang basin filled with Cretaceous–Tertiary strata (Fig. 2). It is divided into the northern and southern domains, with the North Tongbai preserving some tectonic units comparable with those of the North Qinling, such as the Kuanping, Erlangping, and Qinling units [5]. The North Tongbai underwent high-temperature (HT) (770–830°C) to ultra-high temperature (UHT) (920–950°C) metamorphism at ∼440–420 Ma [61], and this episode of UHT metamorphism is also documented in the North Qinling [62].

The Guishan and Nanwan units are thought to be the equivalents to the Wuguan and Liuling units in the Middle Qinling, respectively [5]. As a matter of fact, the Guishan Unit manifests itself as a highly sheared mylonitic zone, with the shearing and synchronous metamorphism happening at 316–314 Ma [61,63]. The Devonian Nanwan Unit consists predominantly of low-grade meta-sedimentary rocks, and their detrital zircons display a wide range of U-Pb age groups such as 970, 840–810, 490–480, 445–440 and 410 Ma [39]. The age spectrum is quite similar to that of the Liuling Unit in the Middle Qinling and the Foziling Group in the North Dabie [14,64,65].

The Balifan shear zone separates the North from South Tongbai domains (Fig. 2), and comprises schists, gneisses, and amphibolites. Ductile shearing and metamorphism occurred in the period of ∼256–242 Ma based on ^40^Ar/^39^Ar dating of muscovites [66]. The Huwan shear zone just south of the Balifan zone expresses itself as a granitic mylonite zone, and also encompasses gneiss, quartz schist and numerous high-pressure (HP) metamorphic bodies such as the Xiongdian and Sujiahe eclogites.

The South Tongbai comprises three metamorphic terranes, the Tongbai complex, ultra-high-pressure (UHP) eclogite terrane and blueschist-greenschist terrane, respectively (Fig. 2). The Tongbai complex experienced high-grade metamorphism in the Late Triassic [5], and had not been exhumed to the surface until the Early Cretaceous [67]. Tectonic histories of the (U)HP eclogite and blueschist-greenschist terranes are related to Late Permian to Early Triassic deep subduction of the continental crust of South China [5]. The three terranes underwent intense deformation and metamorphism in Late Paleozoic and Early Mesozoic times, contrasting with the NQNTO that was created in the Early Paleozoic. The juxtaposition of the North and South Tongbai domains plausibly resulted from large-scale sinistral left-lateral faulting along the Balifan and Huwan shear zones.

### Overview of Early Paleozoic tectonics

#### Structures

The NQNTO was shaped by prolonged multiple crustal deformations, including contraction, extension and transpression in the Paleozoic and Mesozoic. Tectonic evolution of the orogen has been extensively investigated [9–11,37,47], but it still remains a great challenge to restore its Early Paleozoic configuration.

The Luoluan fault separates the Taowan from Kuanping units (Fig. 2), and expresses itself as a ductile shear zone. ^40^Ar/^39^Ar dating of muscovite and biotite from mylonite gives the age of ∼372 Ma [31]. Mesozoic large-scale south-verging thrusting modified the shear zone, and displaced the Taowan Unit southward over the Kuanping Unit [32]. The Zhuxia fault, delimiting the Qinling Unit on the north (Fig. 2), is characterized by dextral shearing [45] and assumed to take place from 426 to 400 Ma [46]. The Shangdan fault, following the Early Paleozoic suture, expressed itself as a left-lateral ductile shear zone [9,11,32]. Sinistral shearing is shown to be from 426 to 400 Ma [37,46], and became reactivated in the Early Jurassic [68]. Of particular interest is the Wuguan shear zone that is located just south of the Shangdan fault and also manifests itself as a wide mylonitic belt (Fig. 2).

The Shanyang fault is another key fault zone separating the Middle from South Qinling (Fig. 2). It merges into the Shangdan fault zone to the east and presumably extends to connect the Balifan fault in the Tongbai region (Fig. 2). The Shanyang fault contains fault breccia, mylonites, sheath folds and stretching lineations, all of which consistently show sinistral sense of movement. Previous studies apparently overlooked the significance of the Shanyang fault on account of the scarcity of detailed documentation of the fault. It is shown that Devonian sequence in the Middle Qinling is completely different from its equivalent in the South Qinling [60]. The juxtaposition of two distinct domains, the Middle and South Qinling, hints at large-magnitude strike-slip displacement of the Shanyang fault. Timing of the onset of the Shanyang fault is not well constrained but it might have commenced since the Carboniferous. This inference relies on the facts that the Wuguan shear zone north of the Shanyang fault gives metamorphic zircon ages of 321–318 Ma of amphibolites and ^40^Ar/^39^Ar ages of 306–299 Ma of hornblende [55,58].

Of particular significance are the multiple deformations of the Qinling Unit. The first-phase deformation was assumed to be the response to deep subduction of continental crust in association with high-grade metamorphism [69 and references therein]. The second-phase deformation is characterized by orogen-parallel crustal stretching, as evidenced by NWW–SEE-oriented ductile stretching lineations and asymmetric intrafolial ductile shear folds [48]. Timing of crustal stretching is deduced from the ages of granitic plutons that formed in an extensional setting from 460 to 420 Ma [41]. Rapid crustal uplift characterizes the third-phase deformation of the Qinling Unit from 400 to 328 Ma based on the ^40^Ar/^39^Ar cooling ages of hornblende and biotite in gneissic rocks [3,47].

#### Magmatism

Early Paleozoic granitoids of various scales widely occur in the NQNTO. Granitic magmatism can be divided into three phases, 507–470 Ma, 460–420 Ma and 420–400 Ma, respectively [41,70]. The first-phase granites are exclusively present in the Qinling Unit, such as the ∼496 Ma Piaochi pluton [70], and are thought to result from crustal anatexis [41]. The second-phase granites crop out throughout the North Qinling, such as the Huichizi and Sikeshu granitic bodies that are dated at ∼466 Ma and 464 Ma, respectively [41]. Coeval granites also exist in the North Tongbai, such as the Taoyuan and Huanggang granites. The third-phase granitic and mafic magmatism is argued to arise from crustal extension and uplift in the post-orogenic stage [41,54].

#### Metamorphism

Three phases of metamorphism are recognized, prograde UHP eclogite and HP eclogite/granulite metamorphism, low-pressure (LP) granulite facies metamorphism, and amphibolite facies metamorphism on the basis of geochronologic data and clockwise P-T-*t* path of metamorphic rocks [43,69]. The UHP eclogites and HP mafic and acid granulite were first identified at the northern edge of the Qinling unit [71], and then discovered in many parts of the unit [72]. The second-phase metamorphism is marked by retrogression of UHP eclogite as a result of high-rate exhumation from mantle depth to lower–middle crust at 480 to 460 Ma in the North Qinling [43,69,73]. In comparison, the 470–460 Ma granulites, amphibolites, and gneisses in the North Tongbai are instead interpreted as the consequence of crustal thickening, possibly due to the continued collision of the North and South China blocks [5,63,74]. The third-phase metamorphism is characterized by amphibolite facies retrogression at 460–410 Ma [61]. The Late Ordovician–Silurian also witnessed a period of HT–UHT metamorphism, as recorded by mafic and pelitic granulites in the Qinling Unit [75].

### Brief assessment of diverse models

A variety of models have been proposed to restore plate tectonic evolution of the NQNTO based on a huge amount of field, geochemical, and geochronological data (Fig. S1). The first model speculates that most of the North Qinling, including the Danfeng, Qinling and Erlangping units, evolved into a continental island arc as a result of subduction of the Proto-Tethyan oceanic plate south of the North China block [8]. A backarc basin developed and gradually separated the island arc from the South China block. The Proto-Tethyan Ocean closed ∼460 Ma, and led to collision of the island arc with the North China block. The Silurian was a post-orogenic period, and the South Qinling then developed as a remnant basin. The uniqueness of this model is that three distinct units are treated as one unity. This simplification obviously neglects many details of tectonic evolution of the North Qinling orogen. Another salient feature is the southward subduction polarity of the Proto-Tethyan oceanic plate. Unfortunately, little geologic evidence supports the interpretation that the South Qinling acted as a huge backarc basin in the early Paleozoic.

The second model supposes that a branch of the Proto-Tethyan Ocean, called the Shangdan Ocean, separated the North from South China blocks. The Shangdan oceanic plate subducted beneath the North China block ∼515 Ma, generating island arcs and triggering backarc extension [1,10,76]. The Qinling micro-continent, as represented by the Qinling Unit, then drifted away from the NCB as a result of spreading of the backarc basin. The Erlangping Unit is assumed as the products of the backarc basin, which then subducted beneath the island arc since the Late Cambrian and closed at the end of the Ordovician. Subduction of the Shangdan oceanic plate had been continuing till the end of the Silurian.

The third model offers a different view on the origin of the Qinling Unit, which is considered as a micro-continent rifting away from the South China block rather than from the NCB [4]. It follows that two branches of the Proto-Tethyan Ocean developed since ∼800 Ma, separated by the Qinling micro-continent. Intra-oceanic subduction began in Branch 1 of the Proto-Tethyan Ocean during the Early Cambrian, and led to the formation of the Erlangping intra-oceanic arc. Collision of the Qinling micro-continent and Erlangping arc occurred around the Late Cambrian and the continued subduction resulted in HP–UHP metamorphism of the leading edge of the Qinling micro-continent from 500 to 490 Ma. Subduction of Branch 2 of the Proto-Tethyan plate or the Shangdan Ocean had persisted throughout the Early Paleozoic, and the Proto-Tethyan Ocean had not closed until the end of the Devonian [4].

The fourth model treats the Qinling Unit as an exotic micro-continent between two branches of the Proto-Tethyan Ocean [39]. Branch 1 (Shangdan Ocean) subducted beneath the Qinling micro-continent, making it evolve into a volcanic arc and triggering extension of Branch 2 in the Late Cambrian. Intra-oceanic subduction took place in Branch 2 at the end of the Ordovician, and generated the intra-oceanic Erlangping arc. The exotic Qinling terrane, Erlangping intra-oceanic arc and NCB were amalgamated in the Silurian, but subduction of the Shangdan Ocean had been continuing until the Late Permian or Triassic [5].

There are some obvious drawbacks in the existing models. (1) Most of the models concur that the Qinling Unit evolved into a volcanic island arc due to subduction of adjacent oceanic plates. However, no Cambrian subduction-related or arc magmatic rocks have been confirmed in the Qinling Unit. The 500–490 Ma igneous rocks are roughly coeval with peak UHP metamorphism and attributed reasonably to anataxis of the deeply subducted continental crust [41]. (2) The UHP eclogitic bodies are distributed throughout the Qinling Unit, indicating that the whole Qinling Unit must have experienced deep continental subduction [77]. Accordingly, the Qinling unit cannot be taken as an island arc terrane when reconstructing the NQNTO tectonic history. This is an important point that all the models must touch upon. (3) Albeit the Danfeng ophiolitic complex is commonly accepted as remnants of the oceanic crust in the existing models [3 and references therein], few studies dealt with their emplacement mechanism. It remains uncertain whether the Danfeng ophiolites resulted from obduction of oceanic crust (Atlantic-type) or a small marginal basin (supra-subduction zone). (4) All the existing models seem to have completely ignored orogen-parallel strike-slip faulting that must have greatly affected the original tectonic architecture of the Qinling–Tongbai orogen. This ignorance leads to doubts about the plausibility of the proposed two-dimensional models in that the present-day juxtaposition of differing tectonic elements might have little to do with each other in the Early Paleozoic.

## THE WUYI-YUNKAI OROGEN

### Tectonic framework

The WYO comprises the main parts of West Cathaysia and the Jiangnan orogen (Fig. 1), and its areal dimension can be defined roughly by spatial distribution of Early Paleozoic granitoids and compressional structures. The Zhenghe–Dapu fault, a prominent sinistral strike-slip fault, serves as the southeastern limit of the orogen, and separates West from East Cathaysia [78]. This fault zone is also considered to roughly follow an Early Paleozoic suture [23,79,80]. Lin *et al.* [28] argued that the boundary between West and East Cathaysia is the Northwest Fujian fault rather than the Zhenghe–Dapu fault (Fig. 1). The Northwest Fujian fault also manifests itself as a left-slip ductile shear zone, and the highly sheared rock assemblages between the Zhenghe–Dapu and Northwest Fujian faults might represent tectonic mélange resulting from oblique amalgamation of East and West Cathaysia [27]. Lin *et al.* [27] delve into rock assemblages of West and East Cathaysia, and confirm that protoliths of Precambrian rocks in West Cathaysia are dominantly Neoproterozoic (1.0–0.8 Ga) in age, contrasting with Mesoproterozoic protolith ages (1.9–1.8 Ga) of basement rocks in East Cathaysia. Moreover, West Cathaysia was extensively affected by Early Paleozoic magmatism, whereas East Cathaysia contains few Paleozoic geologic records and consists primarily of Mesozoic magmatic rocks [78]. It is thus assumed that East Cathaysia was an exotic terrane derived from the Indosinian orogen in the south, and accreted to West Cathaysia in the Late Triassic when it moved northward [27]. The northwestern limit of the WYO is transitional because Early Paleozoic compressional deformations gradually lose their identity northwestward and magmatic rocks also fade away in the same direction (Fig. 1). Also noticeable is that Early Paleozoic magmatism and metamorphism occurred throughout the WYO, which can hardly be ascribed to island-arc tectonic processes [16]. Mesozoic crustal contraction and extension profoundly influenced the WYO, and were accompanied by prodigious magmatism [18,23,81].

### Overview of Early Paleozoic tectonics

#### The Kwangsian orogeny

**Figure 3.**
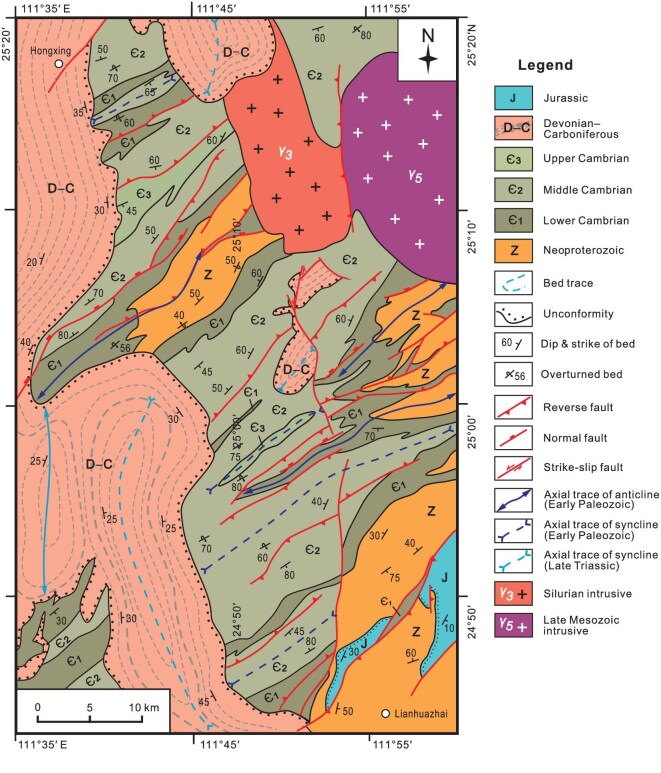
Geological map of the Yongjiang area in West Cathaysia, showing that the Cambrian is intruded by Silurian granite and both of them are overlain unconformably by Devonian–Carboniferous marine deposits. The nonconformity between Silurian granite and Middle Devonian sedimentary rocks indicates that the Wuyi–Yunkai orogen must have experienced broad uplift, erosion and denudation prior to the Middle Devonian.

The Kwangsian orogeny was coined by Ting (1929) who should be credited with identifying an angular unconformity beneath Devonian strata in the Kwangsi (Guangxi) region. This stratigraphic discordance was then recognized throughout West Cathaysia and the Jiangnan orogen (Fig. 3), and a time-equivalent disconformity also exists in the Yangtze block [82]. However, opinions diverge about when the Kwangsian orogeny initiated and how long it persisted. Moreover, geologists have long been plagued by origins of the Kwangsian orogeny and spawned many explanations for the possible tectonic drivers.

**Figure 4.**
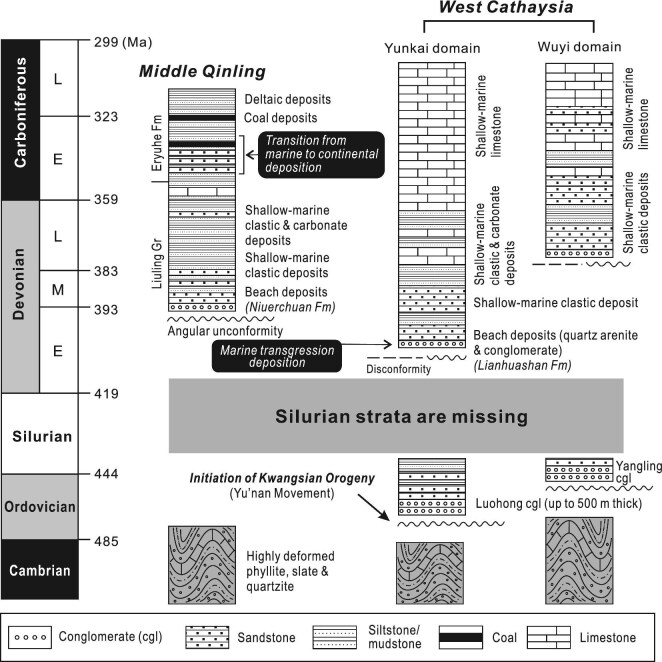
Stratigraphic correlation of the Middle Qinling and the Wuyi and Yunkai domains. Note that stratigraphic sequences of all the three regions are characterized by the presence of an angular unconformity beneath Lower–Middle Devonian strata and the absence of Silurian–Lower Devonian rocks. Another angular unconformity separating the Ordovician from Upper Cambrian occurs in the Yunkai domain, recording an early-stage shortening called the Yu'nan Movement. A marked change of sedimentary environments happened in the Middle Qinling in the Early Carboniferous, with shallow-marine sedimentation giving way to deltaic–fluvial deposition. In comparison, shallow-marine carbonate and siliciclastic deposition continued in the Wuyi and Yunkai areas throughout the Late Paleozoic.

Timing of the onset of the Kwangsian orogeny is basically constrained by paleontological, stratigraphic, and sedimentologic studies [82–84]. Investigations of Lower Paleozoic sequences in the Yunkai domain identify an angular unconformity separating the strongly folded Cambrian strata from the Lower Ordovician conglomerates (Fig. 4), which is assumed to record the first episode of the Kwangsian orogeny in West Cathaysia and called the Yu'nan Movement in Chinese literature [85,86]. Paleontological and stratigraphic studies further show that uplifting of West Cathaysia renewed in the Mid–Late Ordovician and propagated northwestwards [84]. Middle Devonian strata rest unconformably over pre-Devonian units of different ages, with the stratigraphic discordance being expressed as angular unconformities in West Cathaysia and the Jiangnan orogen and disconformities in the Yangtze block [82]. These observations implicate that tectonic push should come from the southeast [83,84]. Silurian strata are basically absent in the interior of West Cathaysia, but present in its peripheral zone including the Jiangnan orogen and southeastern Yangtze block [18]. It is postulated that Silurian sediments were deposited in foreland basins in response to the folding and thrusting in the WYO [18]. It is also suggested that crustal shortening should come to an end before the Middle Silurian based on stratigraphic analysis [86]. Recent studies argued that the Kwangsian orogeny must have ceased prior to the Silurian [27]. End-Ordovician termination of shortening is testified by an unconformity between the deformed Cambrian–Ordovician sequences and the undeformed ∼436 Ma volcanic and pyroclastic rocks in the southwestern WYO [87].

#### Structures

Mesozoic deformations led to significant modification of Early Paleozoic architecture of the WYO [18,88,89]. In general, the Early Paleozoic framework of the WYO was created by two phases of crustal contraction. The first-phase deformation happened at the end of the Cambrian, leading to a NW-verging fold-thrust system [85]. The resulting structures are preserved in the northern Yunkai domain, and manifested by the Damingshan–Dayaoshan fold-thrust belt [86]. This phase of shortening is also registered by a prominent angular discordance separating Cambrian marine sandstone from Lower Ordovician polymict conglomerate [86]. The second-phase deformation took place in the Mid–Late Ordovician, and gave rise to folding, thrusting, and strike-slip faulting throughout the WYO [89]. Li *et al.* [23] argued that sinistral transpression shaped the WYO as a whole, and it was the strain partitioning that led to sinistral shearing along the NE–SW-striking faults and NW–SE contraction. Detailed structural examinations also show that folds and thrusts verge oppositely across the Jiangshao fault, thereby displaying a crustal-scale positive flower structure in cross sections [89].

The Early Devonian witnessed a period of crustal vertical motion. The uplifting event is constrained by cooling ages of muscovite and biotite that yield ^40^Ar/^39^Ar dates from 400 to 397 Ma [23]. Given the absence of Silurian strata in the WYO, surface uplifting might have already started since the Silurian and persisted to the Early Devonian. Crustal vertical motion plausibly led to broad erosion and planation of the WYO based on the observations that Mid–Upper Devonian strata directly overly Silurian intrusives (Fig. 3). The ensuing subsidence then brought about widespread marine inundation and deposition of quartz arenite and limestone during the Mid–Late Devonian.

#### Metamorphism

Early Paleozoic metamorphism is largely in amphibolite to granulite facies grades in the WYO [24]. Geochronologic data of metamorphic zircons converge to indicate that amphibolite and granulite facies metamorphism took place from 460 to 436 Ma [24,90]. Careful studies refine the timespan of different types of metamorphism, with HP amphibolites being formed from 468 to 440 Ma [24,90], and HT/LP granulites from ∼445 to 420 Ma [91]. These data hint at an elevated heat input in the WYO during the Silurian, with geothermal gradients up to 50–56°C/km [91].

#### Magmatism

Late Ordovician–Silurian intrusives are widely distributed in the WYO (Fig. 1), with their ages ranging from 460 to 400 Ma [92]. S-type granites make up ∼90% of the total exposed intrusives, and express them as massive or gneissic plutons [93]. Other types of igneous rocks include I- and A-type granites and mafic rocks [92,94]. There is vigorous debate regarding the origin of granitic magmatism. Prevailing views attribute granitic magma to anataxis of overthickened crust [27,95]. Others argue that granitic magma originated from partial melting of the crust due to heating of the uprising hotter asthenosphere [24]. This argument relies on the observations that 446–410 Ma I-type granite and mafic rocks are closely associated with S-type granite in space and time [96]. Huang and Wang [92] reevaluated different types of granites of the WYO, and concluded that >440 Ma S-type granites formed in a contractional tectonic setting, whereas Silurian–Early Devonian magmatism was generated in an extensional setting in response to delamination of the subcrustal lithospheric mantle and replacement of the hotter asthenosphere.

### Brief assessment of diverse models

Disputes have raged over how the WYO developed, and diverse models are thus advanced on the basis of different approaches (Fig. S2). Two classes of models are now in play for tectonic development of the WYO: one takes it as an intraplate orogen [15,16,18,89] and the other regards it as a collisional one [27,97,98].

Faure *et al.* [26] proposed that the Early Paleozoic orogeny resulted from underthrusting of the West Cathaysia block beneath the Jiangnan orogen along the Jiangshao fault. Intracontinental subduction led to the formation of SE-verging thin-skinned fold-thrust systems over a through-going basal detachment beneath West Cathaysia. It is thought that crustal shortening commenced in the Late Ordovician, culminated in the Silurian, and might have been continuing in the Early Devonian [18,26]. The problems with this model are the improper interpretations of Silurian tectonic settings and ^40^Ar/^39^Ar dates. As described earlier, Silurian igneous rocks formed in an extensional setting rather than in a contractional regime. Given the ^40^Ar/^39^Ar ages for timing of contraction completely overlap the timespan of widespread Silurian high-flux magmatism, the muscovite ^40^Ar/^39^Ar dates possibly represent the cooling ages rather than the timing of folding and thrusting. Li *et al.* [24], instead, proposed that it was the tectonic superposition of thrust sheets that caused crustal thickening of West Cathaysia. The NW-directed thrusting and resultant crustal thickening led to greenschist and amphibolite facies metamorphism of the Nanhua rift successions, and also created foreland basins in front of the thrust system [99]. Silurian magmatism resulted from heating of the rising asthenosphere due to delamination of the subcrustal lithospheric mantle in the post-orogenic period [24,92]. This model satisfactorily accounts for the close association of silicic and mafic magmatism as well as HT/LP metamorphism in Silurian times, and has gained wide acceptance.

The third model is built on careful mapping and analysis of Early Paleozoic structures in the Wuyi domain. The results show that vergence of thrusting in West Cathaysia and the Jiangnan orogen is opposite, with West Cathaysia dominated by SE-directed thin- and thick-skinned folds and thrusts and the Jiangnan orogen by NW-directed thin-skinned tectonics [89]. The tectonic framework was thought of as the result of two-stage crustal contractions: the first-stage deformation was related to north–south compression from 465 to 445 Ma and the second-stage deformation dominated by sinistral transpression from ∼445 to 430 Ma [89].

In contrast to the intraplate models, the WYO is also suggested to arise from continent–continent collision [27,98]. It is assumed that rifting of the Neoproterozoic Nanhua basin led to gradual separation of the Yangtze block and West Cathaysia from 780 to 490 Ma [98]. The resulting oceanic crust then began underthrusting West Cathaysia and closed at ∼460 Ma. It is thought that it was the Yangtze–Cathaysia collision that gave rise to the WYO [91]. Delamination of the subcrustal lithospheric mantle and resultant asthenospheric upwelling combined to bring about extensive granitic and mafic magmatism, high-grade metamorphism, and surface uplifting of the WYO in the Silurian. However, evidence for the existence of Early Paleozoic oceanic crust between the Yangtze block and West Cathaysia remains sparse and nonexistent during the Cambrian and Early Ordovician [100]. It is also postulated that the WYO originated from the collision of West Cathaysia with an exotic block [28].

A chief drawback of all the palinspastic reconstruction is the neglect of Late Paleozoic–Mesozoic clockwise rotation of the South China block, which has been confirmed by both paleomagnetic and geologic studies [13,101]. If the rotational displacement is taken into account, the blocks, which are now juxtaposed with the WYO, would have little to do with Early Paleozoic crustal deformation of the Kwangsian orogeny. It remains a contentious issue to figure out the potential block that once collided with West Cathaysia and created the Early Paleozoic architecture of the WYO.

## CONNECTION OF THE NQNTO AND WYO IN THE EARLY PALEOZOIC

It has long been held that the NQNTO and WYO are two independent Early Paleozoic orogens. The NQNTO resulted from the collision of the North and South China blocks [1], whereas the WYO was an intracontinental orogen [24,88] or an orogen related to the collision of West Cathaysia with an unknown block [28]. The NQNTO boasts a wide range of geologic records of active continental margin, such as ophiolitic assemblages and subduction- and collision-related metamorphism and magmatism [3], but lacks attendant foreland fold-thrust belts and basins. By contrast, the WYO is built up largely by fold-thrust systems and compressional basins, but is devoid of subduction-related ophiolites [16,18]. Notwithstanding the discrepancies, there are many similarities shared by the two orogens. The NQNTO and WYO both experienced crustal shortening from the Late Cambrian to Mid–Late Ordovician (∼510–460 Ma). The Qinling Unit in the NQNTO underwent peak HP–UHP metamorphism at ∼510–490 Ma as a result of deep continental subduction during arc-continental collision [69,77]. This tectonic event coincided with Late Cambrian thrusting and folding in the northern Yunkai domain of the WYO [24,85], and was also registered by an angular unconformity beneath Ordovician strata (Fig. 4). Crustal shortening persisted in both the NQNTO and WYO during the Ordovician, leading to granulite facies metamorphism [24,63,74].

Another concordance of the NQNTO and WYO is the simultaneousness of granitic and mafic magmatism in the Silurian. Around 440–420 Ma, granites intruded the Qinling, Erlangping, Danfeng and Kuanping units (Fig. 2), and concurrent granitic and mafic rocks are distributed throughout the WYO (Fig. 1). In addition, an angular unconformity, separating Devonian strata from underlying units, is evident in both the Middle Qinling and WYO, with Silurian strata being completely missing (Fig. 4). The similar Paleozoic stratigraphic sequences attest to the links between the NQNTO and WYO and hint that both of them were affected by the Kwangsian orogeny.

The discrepancies and likenesses imply that the NQNTO and WYO might have been the integral parts of a dismembered Early Paleozoic orogenic belt. The NQNTO makes up the inner part of the orogen, consisting of ophiolite complexes and subduction- and collision-related metamorphic rock assemblages. The WYO is constructed by fold-thrust systems and foreland basins, representing the outer part of the orogen. The Middle Qinling is a relic of the foreland fold-thrust system, which is still attached to the NQNTO. The two orogens can well match up when united into a single collisional orogen. The connection of the NQNTO and WYO is also justified by paleomagnetic studies that reveal continuous clockwise rotation of the SCB since the late Paleozoic [101]. The eastern WYO should have faced the NQNTO before its departure by means of clockwise rotation. It is estimated that clockwise rotation of the SCB could be more than 67° in the timespan from the late Paleozoic to Triassic [101,102]. Gilder and Courtillot [103] used late Mesozic paleomegnetic data to show that the SCB continued rotating, with its anglar rate being up to 1°/Ma, till the final collision of the North and South China blocks in the Late Jurassic. Consequently, the amount of the SCB clockwise rotation must be ∼100° from 245 to 145 Ma or from the Middle Triassic to Late Jurassic. Geologic studies further demonstrate that the SCB clockwise rotation persisted in the Early Cretaceous [13]. Taken together, the amount of cumulative clockwise rotation must be over 170° from the Permian to Early Cretaceous.

Investigations of detrital zircons from Devonian sandstones also testify to the original connection of the NQNTO and WYO. The Mid–Upper Devonian Liuling Unit in the Middle Qinling contains detrital zircons with a prominent population at 500–400 Ma, two subordinate age clusters at 850–700 Ma and 1000–900 Ma, and a minor group at 2600–2450 Ma [3,56]. Interestingly, detrital zircon grains from Devonian strata in West Cathaysia display quite similar age spectra. Detrital zircons from Lower–Middle Devonian sandstones in the Qinfang basin, located in the southwest of the Yunkai domain, exhibit one conspicuous age peak at 470–400 Ma, two subordinate clusters at 871–700 Ma and 1010–900 Ma, and a minor population at 2600–2400 Ma [104]. The Upper Devonian (Taoziken Fm) in the Yong'an basin in the easternmost West Cathaysia also produces detrital zircons that exhibit one striking age peak at 450–402 Ma and two subordinate clusters at ∼825 Ma and ∼1000 Ma [105]. The identical age spectra of detrital zircons from Devonian sandstones in the Middle Qinling and eastern West Cathaysia confirms the connection of the WYO and Middle Qinling during the Devonian.

Also noteworthy is that Devonian rocks in both the South Qinling and northwest Yangtze block display distinct detrital zircon age spectra. They exhibit two prominent populations at ∼975 Ma and 550 Ma, but are devoid of zircon grains of Late Ordovician and Silurian ages [106,107]. Accordingly, the South Qinling should not be juxtaposed to the Middle Qinling in Devonian time. Meng *et al.* [13] demonstrated that the South Qinling was displaced to its present position along a crustal sinistral shear zone, the Ningshan fault, in the late Mesozoic possibly as a result of the SCB clockwise rotation.

## DEPARTURE OF THE WYO FROM NQNTO

It is inferred that the WYO moved away from the NQNTO since the Carboniferous, and this inference relies on the following lines of evidence:

1. The angular unconformity separating the Lower from Upper Paleozoic strata in the WYO is well correlated with that in the Middle Qinling south of the NQNTO, and characterized by the absence of Silurian strata (Fig. 4). Moreover, Mid–Upper Devonian units in the two regions share similar depositional sequences dominated by neritic siliciclastic and carbonate rocks (Fig. 4) and possess identical detrital zircon age spectrums. Sedimentary facies assemblages then displayed spatial differentiations in the Carboniferous. Continental deposition began in the Middle Qinling since the Carboniferous, contrasting with continuation of shallow-marine sedimentation in the WYO (Fig. 4). Differentiation of facies associations implicates that the NQNTO and WYO had been separated since the Carboniferous and their depositional environments were then under the control of differing processes.
2. Both the Shangdan and Wuguan fault zones began sinistral ductile shearing in the Carboniferous. Biotite ^40^Ar/^39^Ar ages of 323–314 Ma defined the timing the Shangdan strike-slip faulting [32,48], and the onset of the Wuguan sinistral shear zone is constrained by metamorphic zircon ages of 321–318 Ma and hornblende ^40^Ar/^39^Ar ages of 306–299 Ma [55,58]. There is no document of precise ages of the Shanyang shear zone, but its eastern extension, the Balifan shear zone in the northern Tongbai orogen, yield the hornblende ^40^Ar/^39^Ar ages from 316 to 304 Ma [5,63]. The ^40^Ar/^39^Ar dates indicate that the WYO might have begun its departure from the NQNTO since the Carboniferous. Given that the Middle Qinling had never been separated from the NQNTO, the WYO must have moved away along the Shanyang–Balifan shear zone. It is suggested that the Zhenghe–Dapu or the Northwest Fujian shear zone was the trace of the Balifan shear zone (Fig. 1), and records the lateral movement of the WYO because they are all typified by marked sinistral shearing. Lin *et al.* [27] treated the belt between the Zhenghe–Dapu and Northwest Fujian faults as a mélange zone resulting from Late Triassic amalgamation of East and West Cathaysia. We instead argue that this tectonic ‘mélange belt’ was created as a consequence of sinistral transpression related to lateral movement of the SCB since the Late Carboniferous. We build our argument on the timing of differentiation of Carboniferous stratigraphy and sedimentation between the Middle Qinling and WYO.
3. Paleomagnetic studies demonstrate that the South China block started clockwise rotation in the Late Paleozoic [101], leading to the rotational separation of the WYO from NQNTO, together with the SCB. The resulting crustal-scale sinistral shear zone between the NQNTO and WYO was thus sliced apart into two zones, the Shanyang–Balifan and the Zhenghe–Dapu/Northwest Fujian shear zones.

## RECONSTRUCTION OF A BROKEN EARLY PALEOZOIC OROGEN

**Figure 5.**
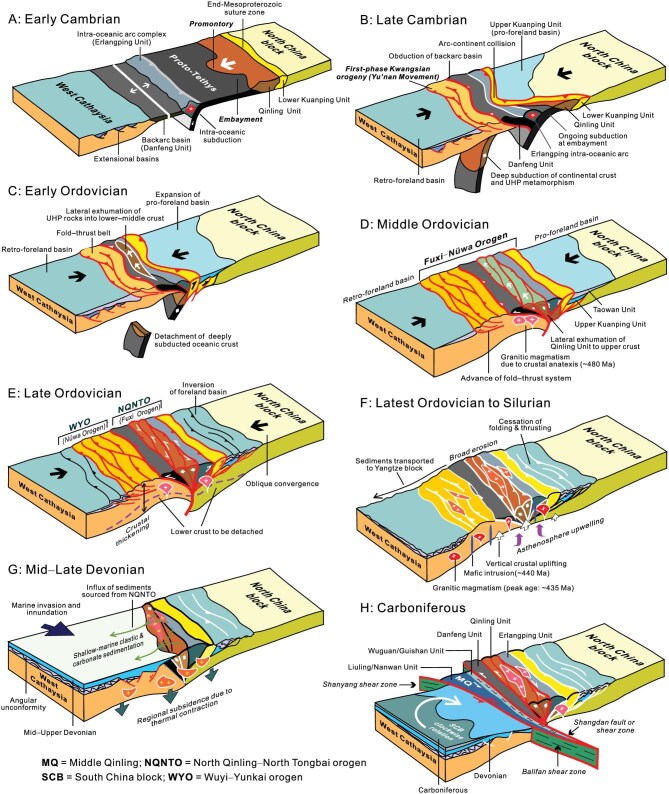
Cartoon diagrams showing how the Fuxi–Nüwa orogen developed. (A) Intra-oceanic subduction occurred in the Proto-Tethyan Ocean south of the North China block (NCB), and created the Erlangping intra-oceanic arc and Danfeng backarc basin in the Early Cambrian. (B) One continental promontory collided with the Erlangping arc, leading to ultra-high-pressure metamorphism, obduction of the Danfeng Unit over West Cathaysia (WC), and creation of fold-thrust belts. (C) Deeply subducted oceanic crust foundered, triggering lateral exhumation of eclogized continental crust toward adjacent embayment. (D) The Fuxi-Nüwa orogen formed due to persistent NCB–WC convergence in Middle Ordovician. (E) Continued convergence thickened the crust/lithosphere of the orogen, and led to outward expansion of fold-thrust belts and foreland basins. (F) Crustal contraction ceased toward the end of the Ordovician, and foundering of subcrustal lithosphere mantle brought about upwelling of the hot asthenosphere. Rising and heating of the asthenosphere triggered silicic and mafic magmatism across the whole orogen. (G) The Mid-LateDevonian witnessed a period when the Fuxi-Nüwa orogen collapsed and thermal contraction of the heated crust/lithosphere cuased broad subsidence and marine invasion. A regional angular unconformity resulted beneath the Devonian across the WYO. (H) Clockwise rotation of the South China block led to various-scale sinistral shearing that eventually dismembered the Fuxi-Nüwa orogen. The WYO gradually moved away from the NQNTO along the Shanyang–Balifan shear zone since the Carboniferous.

Comparative studies of the NQNTO and WYO reveal that the two disparate orogens were once two integrate parts of an enormous orogenic belt that combined the North and South China blocks in the Early Paleozoic. This huge orogenic belt is here named the Fuxi–Nüwa orogen. Fuxi and Nüwa are a god and a goddess in Chinese ancient mythology, and usually portrayed as a couple who intertwined their lower limbs together to form a conjoined twin before their separation. Figure 5 illustrates how the Fuxi–Nüwa orogen evolved in the Early Paleozoic and then broke apart into the NQNTO and WYO in the Late Paleozoic.

The Proto-Tethys separated the NCB from West Cathaysia, and the southern NCB behaved as a passive continental margin (Fig. 5A). Intra-oceanic subduction began around the Early Cambrian, and created the Erlangping intra-oceanic arc and the Danfeng backarc basin. Geologic records along the NQNTO implicate substantial along-strike variations in geometry and tectonic histories of different segments of the orogen [3–5,108]. We infer that the NCB southern margin was originally shaped by promontories and embayments. One of the continental promontories, as represented by the eclogite-bearing Qinling Unit, collided first with the Erlangping intra-oceanic arc in the Late Cambrian when adjacent oceanic plates continued subducting at embayments. Deep subduction of the continental promontory not only resulted in ultra-high-pressure metamorphism of the Qinling Unit, but also caused obduction of the Danfeng backarc basin over West Cathaysia as well (Fig. 5B). As a result, the Danfeng Unit is plausibly the supra-subduction zone ophiolite, and shares emplacement mechanisms similar to many ancient and modern ophiolites, as exemplified by a number of studies [109–111]. The arc–continental collision and oceanic backarc basin obduction brought about crustal shortening at the edge of West Cathaysia in the Late Cambrian, thereby initiating the Kwangsian orogeny. Deeply-subducted oceanic crust was detached and the eclogized continental rocks, or the Qinling Unit, began exhuming laterally to the lower–middle crust toward the west (Fig. 5C). The Fuxi–Nüwa orogen was built up as a result of complete collision of the NCB with West Cathaysia in the Middle Ordovician, and fold-thrust belts and compressional basins developed on its both sides. The eclogized Qinling Unit might have exhumed to the upper crust, and became sandwiched between the Erlangping and Danfeng units (Fig. 5D). S-type granitic magmatism occurred simultaneously due to crustal anatexis. Continued convergence between the NCB and West Cathaysia brought about crustal thickening of the orogen and basin inversion in the Late Ordovician (Fig. 5E).

Subcrustal lithospheric mantle of the overthickened Fuxi–Nüwa orogen was delaminated in the interval from the Late Ordovician to Silurian, thereby bringing about upwelling of the hot asthenosphere (Fig. 5F). A great amount of magma was thus generated due to heating of the uprising asthenosphere, as manifested by widespread concurrences of silicic and mafic intrusions. Uprising of the asthenosphere also caused broad surface uplift of the Fuxi–Nüwa orogen, and led to extensive erosion and possibly planation. The eroded sediments were transported far to the surrounding regions such as the Jiangnan belt and eastern Yangtze block. Magmatism came to an end during the Mid–Late Devonian, and thermal contraction of intrusive bodies led to broad low-rate subsidence of the surface. There then followed shallow-marine sedimentation across West Cathaysia, with the NQNTO serving as one of main provenances (Fig. 5G). Sinistral transpression began affecting the Fuxi–Nüwa orogen since the Carboniferous, and eventually led to disintegration of the orogen into the NQNTO (or the Fuxi orogen) and WYO (or the Nüwa orogen). The WYO left the NQNTO along the Shanyang–Balifan sinistral shear zone due to clockwise rotation of the whole South China block (Fig. 5H). The Middle Qinling, a part of the WYO Early Paleozoic fold–thrust belt, remained attached to the NQNTO when West Cathaysia moved away. Consequently, the NQNTO largely preserves the subduction- and collision-related tectonic history of the Fuxi–Nüwa orogen, whereas the WYO records the development of a foreland fold-thrust system and foreland basin.

**Figure 6.**
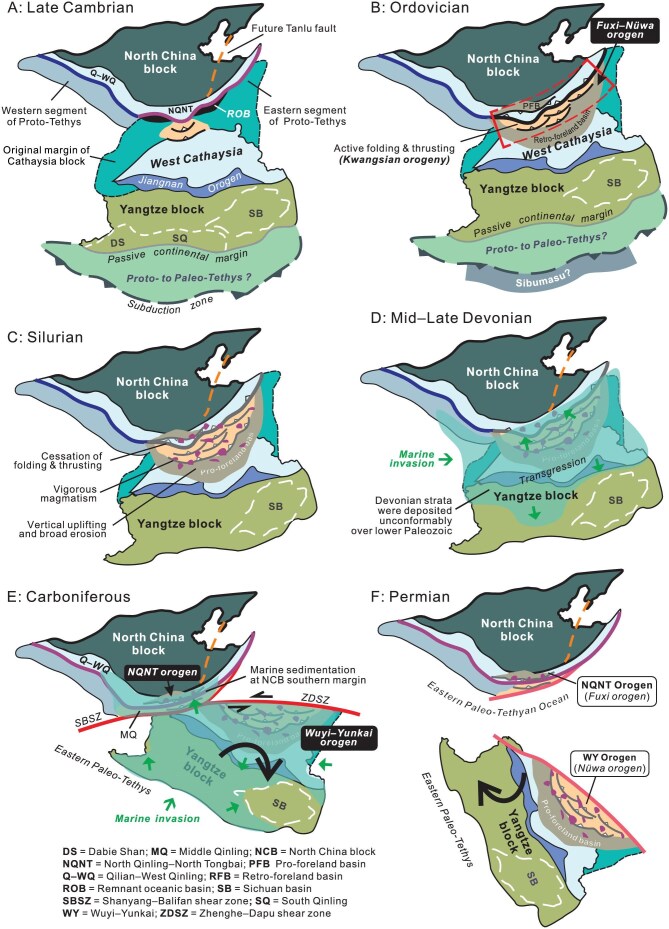
Diagrams showing the creation and fragmentation of the Fuxi–Nüwa orogen in East China in map view. (A) Late Cambrian collision of a promontory of North China block (NCB) with West Cathaysia (WC) led to initiation of folding and thrusting in West Cathaysia. (B) Complete NCB–WC collision took place in the Ordovician and built the Fuxi–Nüwa orogen. (C) Crustal shortening ceased at the end of the Ordovician, and Silurian asthenosphere upwelling caused prodigious magmatism and raised the Fuxi–Nüwa orogen, leading to extensive surface denudation and planation. (D) Mid–Late Devonian marine transgression gave rise to submergence of the peneplaned Fuxi–Nüwa orogen. (E) Clockwise rotation of the South China block (SCB) brought about splitting of the Fuxi–Nüwa orogen since the Carboniferous, which was ripped into the North Qinling–North Tongbai orogen (NQNTO) and Wuyi–Yunkai orogen (WYO) along the Shanyang–Balifan and Zhenghe–Dapu shear zones. (F) The WYO eventually moved away from the NQNTO, with the eastern Paleo-Tethys opening and expanding around the clockwise rotating SCB.

Figure 6 provides a panorama showing how the Fuxi–Nüwa orogen was formed and then dismembered into the NQNTO and WYO in map view. Initial collision of the NCB with West Cathaysia took place at a promontory of the NCB southern margin in the Late Cambrian, and led to folding and thrusting in the Yunkai domain of the WYO. Subduction of oceanic plate continued at adjacent embayments. There might have developed another Proto-Tethys south of the Yangtze block that acted as a passive continental margin at that time (Fig. 6A). Persistent convergence resulted in complete collision of the two blocks during the Ordovician, and constructed the Fuxi–Nüwa orogen (Fig. 6B). Meanwhile, the Proto-Tethyan branch south of the Yangtze block continued subducting beneath the terranes along the northeast margins of East Gondwana, possibly the Sibumasu or Indochina (Fig. 6B). Crustal shortening had come to an end before the Silurian, and high-flux magmatism then prevailed throughout the Fuxi–Nüwa orogen (Fig. 6C). Crustal vertical motion facilitated broad erosion, and planation of the orogen during the period from the Silurian to Early Devonian. The Mid–Late Devonian saw a period of widespread marine invasion into West Cathaysia, bringing about transgressive deposition of shallow-marine quartz arenite and neritic carbonate rocks over the underlying strata (Fig. 6D). Extensive marine inundation was a combined consequence of planation and thermal subsidence of the Fuxi–Nüwa orogen in the aftermath of Silurian erosion and magmatism. The Fuxi–Nüwa orogen was then cut into two during the Carboniferous owing to large-magnitude lateral movement of the SCB (Fig. 6E). The Shanyang–Balifan and Zhenghe–Dapu/Northwest Fujian shear zones serve as the tectonic boundary of the two separated components, the NQNTO and WYO. The Middle Qinling was still attached to the NQNTO. The SCB continued rotating clockwise and gradually drifted away from the NCB, leading to the creation of the eastern branch of the Paleo-Tethyan Ocean (Fig. 6F). Continuous clockwise rotation of the SCB eventually brought about a scissor-like collision of the Yangtze block and the NCB in the Mesozoic [101,103].

Restoration of the dismembered Fuxi–Nüwa orogen is of great significance for reshaping our understanding of the Proto-Tethyan evolution of East China. The proposed tectonic scenarios can well account for many conundrums that have plagued geologists working on tectonics of the NQNTO and WYO for decades. The key problems include: (1) the driver for the Kwangsian orogeny in West Cathaysia; (2) the concurrence of Silurian–Early Devonian silicic and mafic magmatism in both the NQNTO and WYO; (3) the causes for the absence of Silurian strata throughout the NQNTO and WYO; and (4) the origins for Mid–Late Devonian marine inundation over West Cathaysia. This study offers a plausible solution to these enigmas. First, it is proposed that collision of the NCB with West Cathaysia created the Fuxi–Nüwa orogen, with the WYO constituting its foreland fold-thrust system and foreland basin. It was thus the collision of the NCB with West Cathaysia that drove the Kwangsian orogeny. Second, Silurian high-flux magmatism in the NQNTO and WYO was previously taken as independent thermal–tectonic events, and attributed to differing tectonic processes. Our model supposes that prodigious silicic magmatism and attendant mafic intrusion were generated by heating of the rising asthenosphere due to foundering of the subcrustal lithospheric mantle of the Fuxi–Nüwa orogen. This surmise is compatible with the previously-proposed trigger for Silurian magmatism in the WYO [24]. Third, the absence of Silurian strata arose from broad vertical surface uplift and concomitant denudation as a result of the asthenospheric upwelling, which affected the whole Fuxi–Nüwa orogen. Fourth, extensive deposition of Mid–Late Devonian quartz arenites and limestones suggests broad low-rate subsidence of West Cathaysia, which can be readily attributed to thermal contraction of the Silurian plutons and hotter asthenospheric materials. The resulting extensive marine incursion can also account for the presence of Upper Devonian–Carboniferous marine and paralic deposits in the southern edge of the eastern NCB (Fig. 6D and E), which has been a bewildering problem for the existing tectonic models to tackle [112,113].

Another important assumption of this study is the irregular margin architecture of the NCB, expressed as alternating promontories and embayments. The conjecture that the NCB promontories and embayments successively collided with the Erlangping arc terrane can satisfactorily explain some perplexing problems with tectonics of the NQNTO. (1) The continental promontory–arc collision took place first in the Late Cambrian, and facilitated deep subduction of continental crust, thereby leading to UHP metamorphism of the Qinling Unit. Oceanic plate subduction continued at adjacent embayments and complete collision happened toward the end of the Ordovician. (2) The model provides a good solution to lateral rapid exhumation of the eclogite-bearing Qinling Unit at ∼490–480 Ma in that adjacent embayment could serve as an easy route for deeply-subducted continental crust at promontories to go upward laterally. (3) The first-phase crustal shortening of the WYO or the onset of the Kwangsian orogeny in the Yunkai domain coincided with the continental promontory–arc point collision in the North Qinling.

A puzzling problem is what happened to the Yangtze craton when the convergence was going on between the NCB and West Cathaysia. A number of studies assume that there developed another branch of the Proto-Tethys, called the southern Proto-Tethys [25], which separated the SCB from the terranes located along the northeast margin of East Gondwana, such as the Sibumasu, Indosinian and North Qiangtang [114]. The southern Proto-Tethyan branch is argued to subduct beneath the terranes [115,116], with the Yangtze craton behaving as a passive continental margin (Fig. 6A and B). This inference is borne out by continuous marine sedimentation and rift basin development in the Yangtze block in the Early Paleozoic, as demonstrated by both geologic observations and seismic profiles [2,117]. In addition, the South Qinling, which was then located in the leading edge of the Yangtze craton, was characterized by occurrences of ∼481–399 Ma alkaline and intermediate–mafic rocks that indicate an extensional tectonic setting [118,119]. Liu *et al.* [117] argued that the southern Proto-Tethys might have experienced complicated evolution, such as flipping of subduction polarity and formation of several backarc basins. The southern branch of the Proto-Tethys might have persisted into the Late Paleozoic and possibly had not completely closed till the Late Triassic.

The reconstruction of the Proto-Tethyan tectonics in East China is also of significance for hydrocarbon exploration in the SCB. It has been shown that Lower Paleozoic strata in the Yangtze block possesses great petroleum potential, and the Lower Cambrian Qiongzhusi, Upper Ordovician Wufeng and Lower Silurian Longmaxi formations turn out to be excellent source rocks for unconventional hydrocarbons [120–122]. It used to be regarded that Early Paleozoic stratigraphic and sedimentologic development of the Yangtze block was closely related to the Qinling tectonics [76,123]. This study, however, shows that the Yangtze block, including the South Qinling, was located far from the North Qinling orogen in the Early Paleozoic (Fig. 6A–C). Behaving as a passive continental margin of the southern Proto-Tethyan branch, the Yangtze block provided favorable tectonic and sedimentary environments for deposition of superb source rocks, and thus the Lower Paleozoic there should have excellent hydrocarbon perspectives.

## CONCLUSIONS

Holistic treatment of two separate Early Paleozoic orogens, the NQNTO and WYO, led to the discovery of an enormous orogenic belt that once existed in East China, named here the Fuxi–Nüwa orogen. Our new findings are summarized as follows: (1) the NQNTO and WYO were two integral parts of the Fuxi–Nüwa orogen, with the former consisting mostly of subduction- and collision-related rock assemblages and the later made up of foreland fold-thrust systems and foreland basin; (2) tectonic evolution of the Fuxi–Nüwa orogen involved intra-oceanic subduction, continent-arc collision, and backarc basin obduction; (3) UHP metamorphism resulted from deep subduction of a continental promontory, and exhumation of the eclogite-bearing Qinling Unit was achieved by lateral extrusion at an adjacent embayment; (4) it was the collision of the NCB with West Cathaysia that drove the Kwangsian orogeny, and crustal shortening had ceased prior to the Silurian; (5) upwelling of the asthenosphere triggered Silurian vigorous magmatism and caused extensive surface uplift and erosion, which led to the missing Silurian strata across the whole Fuxi–Nüwa orogen; (6) the Fuxi–Nüwa orogen was sliced into two, the NQNTO and WYO, due to lateral movement of the SCB along the Shanyang–Balifan shear zone since the Carboniferous. The WYO, together with the SCB, then began its Paleo-Tethyan evolution in the Late Paleozoic.

## Supplementary Material

nwaf153_Supplemental_Files
