# Supplementary material for: Proto-Tethyan tectonics in East China: a revisit
Source: Natl Sci Rev. 2025 Apr 21;12(6):nwaf153. doi: 10.1093/nsr/nwaf153 (PMC12121481; doi:10.1093/nsr/nwaf153)
Supplement: nwaf153_Supplemental_Files [file nwaf153_supplemental_files.zip › Figure S1.pdf]

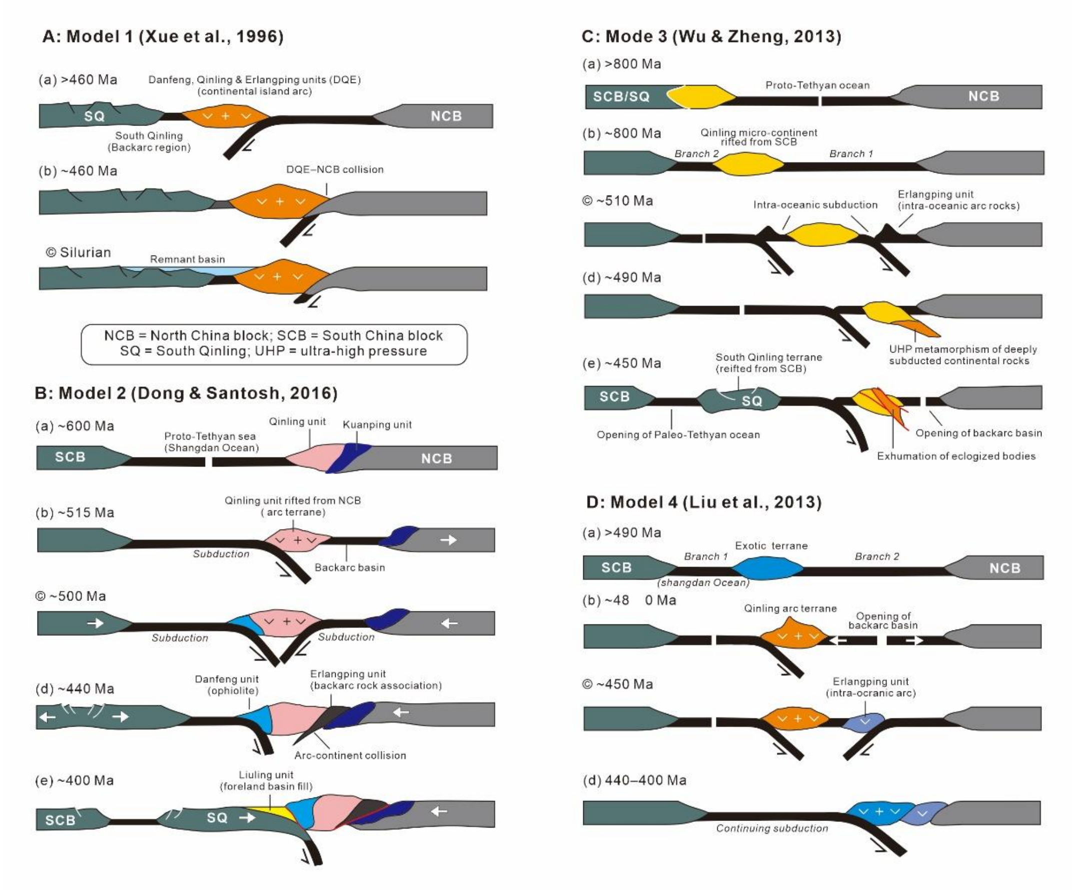

Figure S1. Summary of several proposed 2D tectonic models for the North Qinling–North Tongbai orogen. Refer to the text for detailed assessment of the palinspastic reconstructions.
