# Supplementary material for: Proto-Tethyan tectonics in East China: a revisit
Source: Natl Sci Rev. 2025 Apr 21;12(6):nwaf153. doi: 10.1093/nsr/nwaf153 (PMC12121481; doi:10.1093/nsr/nwaf153)
Supplement: nwaf153_Supplemental_Files [file nwaf153_supplemental_files.zip › Figure S2.pdf]

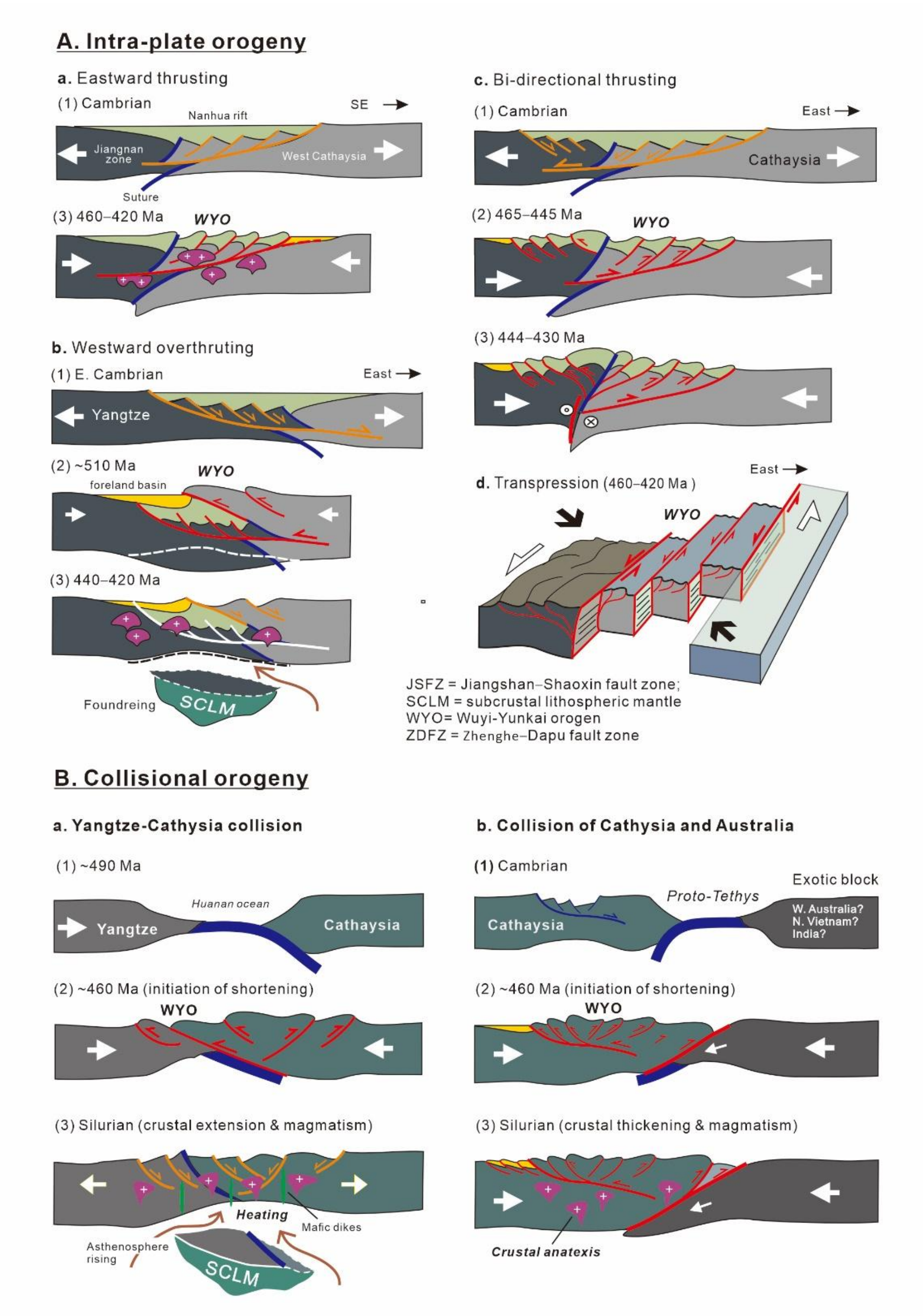

Figure S2 Summary of main proposed models for tectonic evolution of the Wuyi–Yunkai orogen, which can be divided into two categories, intra-plate and collisional orogens, respectively. Refer to the text for detailed assessment of the palinspastic reconstructions.
